# Ablation apprentices and their first experience of pulmonary vein isolation procedure on paroxysmal atrial fibrillation with different sheaths

**DOI:** 10.1186/s13019-024-02826-4

**Published:** 2024-07-13

**Authors:** Ye Liu, Jingjing Song, Siyu Wang, Lifeng Liu, Xiaoqing Liu, Zheng Liu, Yuxing Wang, Lei Zhao, Xinchun Yang

**Affiliations:** Heart Center & Beijing Key Laboratory of Hypertension, Beijing Chaoyang Hospital, Capital Medical University, Beijing, 100020 China

## Abstract

**Objectives:**

This study aimed at exploring how using different kinds of sheaths will affect the very first ablation procedure of apprentices.

**Methods:**

15 patients with paroxysmal atrial fibrillation were randomized to used fixed-curve, conventional steerable or visualized steerable sheath, and received complete isolation of pulmonary veins. All ablations were the very first procedure performed by 15 ablation apprentices. The use of fluoroscopy and catheter stability during the PVI were analyzed.

**Results:**

Procedure duration was much longer in the fixed-curve group (116.8 ± 27 vs. 62.2 ± 17 vs. 60.4 ± 17, *p* < 0.001). X-ray exposure was lowest with visualized sheath (17.6 ± 5 vs. 18.6 ± 6 vs. 5.2 ± 6, *p* < 0.001). CF SD differed significantly, especially at the anterior aspect of LSPV (7.90 ± 2.90 vs. 5.04 ± 2.18 vs. 4.52 ± 2.40, *p* < 0.001) and posterior aspect of RSPV (6.84 ± 2.79 vs. 3.42 ± 2.04 vs. 3.50 ± 2.30, *p* < 0.001) in the fixed-curve group. Impedance drop was significantly smaller in the fixed-curve group at the anterior aspect of LSPV (8.74 ± 3.02 vs. 11.49 ± 5.48 vs. 12.57 ± 5.96, *p* = 0.005).

**Conclusion:**

Even for the very first ablation procedure of an ablation apprentice, the use of steerable sheaths will significantly reduce the procedure duration and improve the catheter stability, but only visualized steerable sheath can reduce fluoroscopic time.

## Introduction

Catheter ablation has become the first line therapy to treat paroxysmal atrial fibrillation (AF) [1]. Success rate continues to rise as the advancement of sophisticated tools and 3D navigation system. The key to ablation of paroxysmal AF is success pulmonary vein isolation (PVI) [1]. During recent years, numerous technological developments in the domain of catheter design, 3D anatomic orientation, catheter navigation, and catheter contact have been introduced to facilitate AF ablation procedures [2–4]. Various sheaths with different shapes and sizes have been used to support the ablation catheters. Steerable sheaths enable the operator to alter the sheath shape and improve manipulation and stability of the catheter around the LA [5]. Now novel 3D visualized sheaths have been developed to reduce the fluoroscopic time during radiofrequency applications. Some studies described that with steerable and visualized sheaths experienced interventionists will reduce fluoroscopic and procedural time for some elements of AF ablation [6, 7]. However, almost all procedures in these studies were performed by highly experienced experts [7–9], and few research focus on those inexperienced apprentices especially with their very first ablation procedure. This observational study aimed to assess the ablation process, fluoroscopic time, catheter stability and success rate of the first experience of PVI with different sheaths.

### Study design

We incorporated 15 apprentices of catheter ablation at Beijing Chaoyang Hospital who had their very first pulmonary vein isolation (PVI) between January 2017 and December 2020. Procedures were guided using CARTO 3 mapping system. All apprentices have n ever performed PVI beforebut were familiar with the technique of ablation, having observed more than 10 procedures and were able to perform vascular and septal puncture and mapping with high density catheters. While none had experience with other types of catheter ablations such as TPSV, four apprentices had prior experience with coronary artery interventions, providing foundational skills beneficial for learning PVI procedures. They were randomized into 3 groups immediately prior to the ablation procedure using pre-prepared randomization envelopes. 5 in the fixed-curve group with Fast-Cath transseptal guiding introducer SL1 (St. Jude Medical Inc., St. Paul, MN, USA). 5 (conventional steerable group) with a conventional steerable sheath (Agilis NxT Steerable Introducer; Abbott Laboratories, Abbott Park, IL, USA) and the remaining 5 used 3D visualized steerable sheath (VIZIGO bi-directional sheath; BiosenseWebster, Diamond Bar, CA, USA). Experienced experts of catheter ablation were acting as supervisor during the procedure. If bi-directional isolation was not achieved after two ablation circles, the supervisor export would take over. The ablation procedure used open-irrigated ThermoCool SmartTouch Surround Flow catheter (Biosense Webster). The observation began when the first ablation point in the left superior pulmonary vein was initiated and terminated when the last ablation point in the right superior pulmonary vein finished. Before undertaking their first ablation procedures, each operator tested all three catheter types—fixed-curve, conventional steerable, and visualized steerable—multiple times. This preliminary testing was conducted to ensure each operator was well-acquainted with the functionality and handling characteristics of each catheter type, which facilitated ease of manipulation due to their unique designs such as the double side curves.”

15 patients with paroxysmal atrial fibrillation who underwent catheter ablation were incorporated in the study. Paroxysmal AF (PAF) was determined as AF episodes that were self-terminating or converted within 7 days. Demographic information and clinical features of all patients were collected from electronic medical records (EMR). Each 5 patients were randomized into fixed-cure, conventional steerable and visualized steerable group using the same randomization envelopes immediately prior to the ablation procedure and received PVI with according apprentice. All patients received informed consent, and the study protocol conforms to the ethical guidelines of the 1957 Declaration of Helsinki as reflected in a prior approval by the Institution’s Human Research Committee.

### Settings before ablation

All antiarrhythmic drugs were discontinued for over five half-lives before the procedure. The study was performed with patients under general anaesthesia. Transesophageal echocardiography were used to exclude thrombus in left atrial appendage. A multislice helical CT was performed for reconstruction of the left atrium. After femoral venous puncture on both sides, an intracardiac echocardiography (ICE) catheter (SOUNDSTAR, Biosense Webster) and a fixed-curve catheter (SL1; St. Jude Medical Inc., St. Paul, MN, USA) were introduced. Transseptal puncture approach was performed under the guidance of fluoroscope and ICE [10]0.100 IU/kg of heparin was administered intravenously immediately after transseptal catheterization. Both ICE and high-density Mapping Catheter (Pentaray NAV, Biosense Webster) were used cooperatively for anatomic mapping of left atrium. Left atrium volume was measured with CARTO 3 system after mapping. In fixed-curve group, the fixed-curve SL1 sheath in first place were continuously used in the following ablation. While in conventional and visualized steerable groups, the original fixed-curve SL1 sheath was replaced with a new steerable sheath accordingly. 56-pore ThermoCool SmartTouch Surround Flow catheter (Biosense Webster) was introduced for the following ablation.

### Catheter ablation

**Fig. 1:**
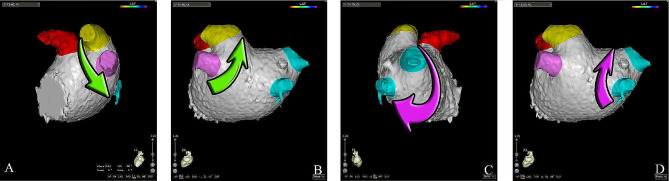
Ablation sequence of the procedure. The procedure started with the anterior ridge of left superior pulmonary vein (**A**, left lateral view) and went counter-clockwise (**B**, posterior view) to achieve isolation of left pulmonary veins. Then the anterior ridge of right-superior pulmonary vein (**C**, right lateral view) and went clockwise (**D**, posterior view) to achieve isolation of right pulmonary veins

An activated clotting time of between 250 and 300 s was maintained by heparin during the procedure. A point-by-point circumferential PVI was performed (illustrated in Fig. 1). The procedure started with the anterior ridge of left superior pulmonary vein and went counter-clockwise to achieve isolation of left pulmonary veins. Then the anterior ridge of right-superior pulmonary vein and went clockwise to achieve isolation of right pulmonary veins. Contact force (CF) was finetuned to 10–15 g and inter-lesion distance (ILD) of < 6 mm with the use of the real-time automated tagging module (VisiTag Module, CARTO3; Biosense Webster). Power output was set to 45 W at a saline irrigation rate of 15mL/min (CoolFlow Pump; Biosense Webster). Radiofrequency was delivered until a target ablation index (AI) 550 at the anterior wall and 400 at the posterior wall/roof [11]. The RF application was stopped once the target AI was reached, and the catheter was maneuver to the next spot. A new ablation was initiated after verifying stability, CF and ILD. If an RF tag not reaching the AI target due to catheter instability, a new RF application reaching the AI target would then be applied in the same spot. Completion of the PVI was confirmed by bidirectional (entrance and exit) block. The task of apprentices ended when two ablation was completed. If the first-pass PVI was not achieved after a complete circumferential ablation, the supervisor would take over and touch-up ablation was delivered until PVI.

### Ablation positions and parameters of PVI

We compared the duration of completion of circumferential ablation, catheter stability, the fluoroscopic time and radiation dose during the ablation procedure. Each ablation point was distributed to the following LA-PV segment. The anterior, posterior, and superior aspects of the right superior pulmonary veins (RSPVs) and LSPVs. Anterior, posterior, and inferior aspects of the right and left inferior PVs (RIPVs and LIPVs, respectively).

All ablation parameters, including contact force (g), power output (W), RF duration (seconds), impedance drop (Δ-Imp, ohms [Ω]), and ablation index (AI) recorded were extracted from the CARTO3 mapping system. The standard deviation (SD) of the contact force (CF SD [g]) was calculated from the CF data recorded every 0.05 s during the ablation. After the beginning of ablation, the fluoroscopic time and radiation dose during the ablation procedure were extracted from the Philips DSA system.

### Follow up

After ablation, patients were carefully monitored in wards for perioperative complications for at least 2 days. Routine follow-up with 24 h ECG was arranged at 3rd and 6th month after the operation. Instant ECG was performed at outpatient clinic when the patient had any relavent symptom. To enhance the precision of our follow-up protocol, we have meticulously documented all follow-up encounters, including any recurrent arrhythmic events and subsequent interventions, up to 12 months post-ablation. This rigorous approach allows us to assess the long-term effectiveness and compare outcomes comprehensively across different sheath groups used in the study.

### Statistical analysis

Continuous variables were presented as mean ± IQR (interquartile range) in demographic characteristics and mean ± SD in parameters of ablation points. Categorical variables are expressed as the number and percentage of patients. Analysis of variance (ANOVA) was used to analyze the differences in the continuous variables, and the chi-square test was used to analyze the differences in the dichotomous variables. SPSS 23 was used for statistical analysis. P-value of < 0.05 was considered statistically significant.

## Results

### Participant characteristics

The study population consisted of 15 patients (11 male, mean age 63.73 ± 10 years) who received their first catheter ablation of paroxysmal atrial fibrillation between January 2017 and December 2020. These ablations were the very first procedures performed by 15 ablation apprentices (8 male, mean age 36.07 ± 3 years). Table 1 summarizes the demographic and clinical features of our study participants, including age, gender, comorbidities, and detailed measurements of left atrium volume post-3D mapping, ensuring a comprehensive overview for a balanced comparison. There were no significant differences in gender, age, LA diameter, LA volume, comorbidity, and use of antiarrhythmic drugs among fixed-curve, conventional steerable, and visualized steerable groups. 5 of apprentices had previous experience of coronary artery intervention.

**Table 1:** Demographic characteristics of participants (doctor apprentices and patients)

	Fixed-curve (*n* = 5)	Conventional (*n* = 5)	Visualized (*n* = 5)	*P* value
**Doctor apprentices**				
Age	35.8 ± 3	35.6 ± 3	36.1 ± 4	0.560
Male (%)	3(60)	3(60)	2(40)	0.765
Had coronary intervention experience (%)	2(40)	2(40)	1(20)	0.741
**Patients**				
Age	62.2 ± 12	64.0 ± 13	65.0 ± 17	0.844
Male (%)	5(100)	4(80)	2(40)	0.231
HTN (%)	1(20)	3(60)	4(80)	0.301
CAD (%)	0	0	1(20)	0.343
DM (%)	1(20)	2(40)	1(20)	0.711
EF (%)	65.6 ± 8	67.2 ± 14	65.2 ± 10	0.855
LA (mm)	40.8 ± 7	40.2 ± 5	41.8 ± 5	0.685
LA volume (ml)	147.2 ± 10.2	143.6 ± 12.5	146.2 ± 11.3	0.820
LA dwelling time (min)	116.8 ± 27*	62.2 ± 17	60.4 ± 17	< 0.001
Ablation duration (s)	2051.8 ± 1075	1995.6 ± 480	1907.4 ± 430	0.846
Ablation point	74.8 ± 14	71.2 ± 15	72.6 ± 22	0.756
Exposure dose (mGy)	17.6 ± 5	18.8 ± 6	5.2 ± 6*	< 0.001
Exposure frequency	18.4 ± 8	20.2 ± 4	5.4 ± 4*	< 0.001

### Procedure duration and fluoroscopic time

As was shown in Table 1. Procedure duration represented as LA dwelling time was much longer in the fixed-curve than the other two (116.8 ± 27 vs. 62.2 ± 17 vs. 60.4 ± 17, *p* < 0.001). Fluoroscopic dose was significantly lower in the visualized sheath group (17.6 ± 5 vs. 18.6 ± 6 vs. 5.2 ± 6, *p* < 0.001). The frequency of exposure was lowest in the visualized group. Conventional steerable sheath needed more exposure frequency, but LSD post-hoc analysis showed no significance (18.4 ± 8 vs20.2 ± 4 vs. 5.4 ± 4, *p* < 0.001).

### Catheter stability

**Fig. 2:**
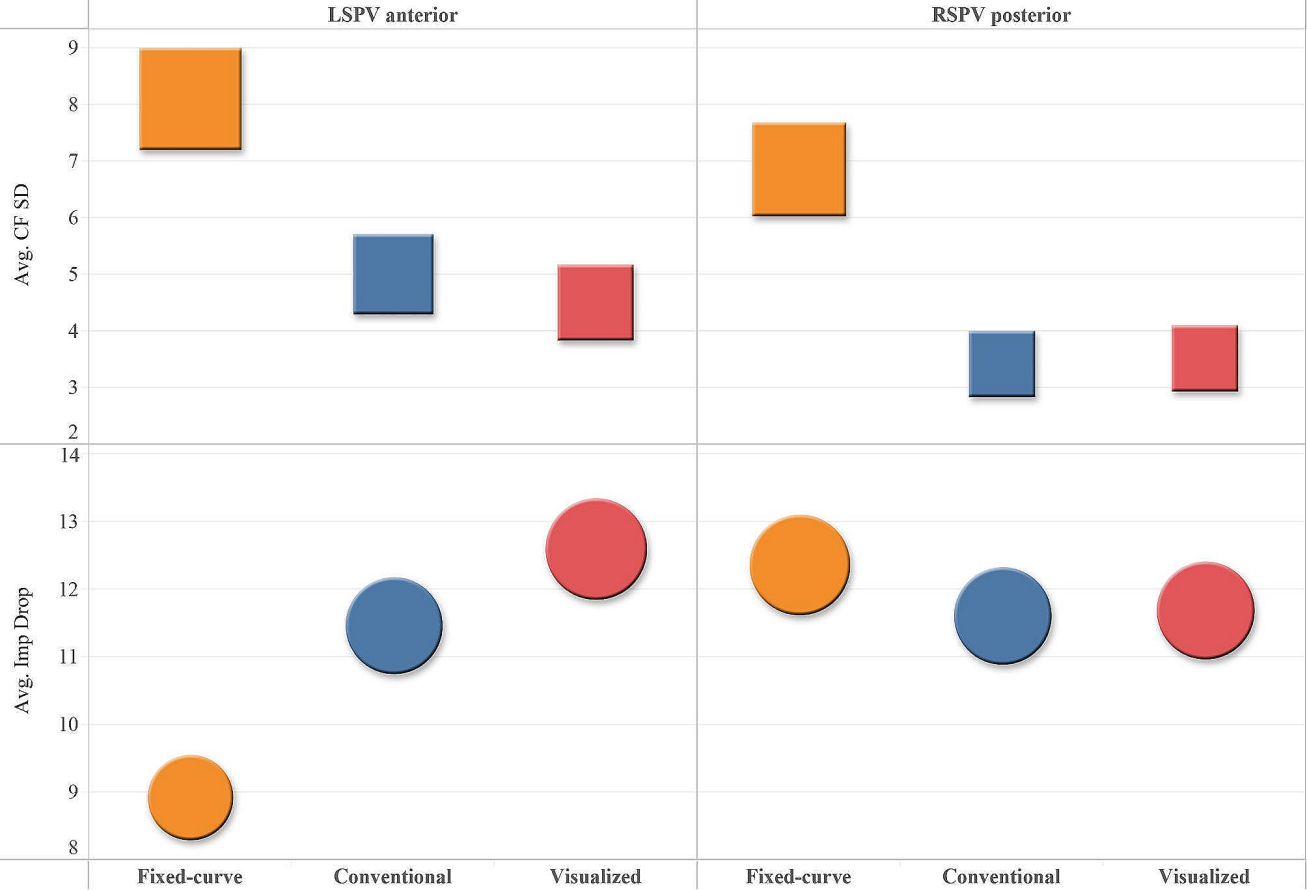
Catheter stability indicated by CF SD and Imp drop at anterior aspect of LSPV and posterior aspect of RSPV. *CF SD, contact force standard deviation; Imp, impedence; LSPV, left superior pulmonary vein; RSPV, right superior pulmonary vein*

1080 ablation points have been recorded (Table 2). The overall AI, CF SD, Δ Imp and CF showed no statistic significance. CF SD was much larger with the fixed-curve group, and was similar between conventional and visualized steerable (5.09 ± 2.73 vs. 4.24 ± 2.40 vs. 4.33 ± 2.37, *p* = 0.001). Larger CF SD occurred in the fixed-curve group, especially at anterior aspect of LSPV (7.90 ± 2.90 vs. 5.04 ± 2.18 vs. 4.52 ± 2.40, *p* < 0.001) and posterior aspect of RSPV (6.84 ± 2.79 vs. 3.42 ± 2.04 vs. 3.50 ± 2.30, *p* < 0.001) (Fig. 2), but post hoc analysis did not show statistical significance in two steerable groups. The impedance drop was significantly smaller in the fixed-curve group at the anterior aspect of LSPV (8.74 ± 3.02 vs. 11.49 ± 5.48 vs. 12.57 ± 5.96, *p* = 0.005) (Fig. 2). No differences were found in other parts of PVs (Table 3).

**Table 2:** Parameters of ablation points

	Fixed-curve (*n* = 374)	Conventional (*n* = 356)	Visualized (*n* = 350)	*P* value
AI	469.22 ± 52.63	471.35 ± 51.33	468.69 ± 53.40	0.775
Δ Imp	11.77 ± 6.49	12.03 ± 7.04	11.89 ± 6.65	0.865
CF	13.80 ± 4.89	13.20 ± 4.80	11.90 ± 4.14*	< 0.001
CF SD	5.09 ± 2.73*	4.24 ± 2.40	4.33 ± 2.37	0.001

AI, ablation index; Δ Imp, impedance drop; CF SD, contact force standard deviation

**Table 3:** Catheter stability and ablation effects at each aspect of pulmonary veins

			Fixed-curve	Conventional	Visualized	*P*
LSPV	anterior	CF SD	7.90 ± 2.90*	5.04 ± 2.18	4.52 ± 2.40	< 0.001
		ΔImp	8.74 ± 3.02*	11.49 ± 5.48	12.57 ± 5.96	0.005
	posterior	CF SD	4.67 ± 2.18	3.93 ± 2.37	4.24 ± 1.76	0.311
		Δ Imp	11.01 ± 6.16	11.00 ± 5.40	11.50 ± 7.92	0.929
	superior	CF SD	4.45 ± 2.51	4.25 ± 2.10	5.02 ± 2.34	0.326
		Δ Imp	12.22 ± 5.90	12.41 ± 7.00	11.92 ± 5.71	0.943
LIPV	anterior	CF SD	4.73 ± 2.41	4.40 ± 2.03	4.86 ± 2.01	0.659
		Δ Imp	11.50 ± 7.02	13.86 ± 7.80	11.28 ± 6.26	0.234
	posterior	CF SD	4.86 ± 2.23	4.64 ± 2.35	4.39 ± 2.39	0.678
		Δ Imp	11.26 ± 6.37	12.90 ± 8.35	12.38 ± 6.14	0.584
	inferior	CF SD	4.59 ± 2.14	4.39 ± 2.90	4.66 ± 2.49	0.894
		Δ Imp	11.19 ± 6.07	12.53 ± 7.56	11.85 ± 6.23	0.684
RSPV	anterior	CF SD	5.52 ± 2.75	4.50 ± 2.90	4.63 ± 2.94	0.261
		Δ Imp	14.49 ± 7.96	11.73 ± 5.70	11.62 ± 7.74	0.164
	posterior	CF SD	6.84 ± 2.79*	3.42 ± 2.04	3.50 ± 2.30	< 0.001
		Δ Imp	12.39 ± 7.15	11.64 ± 7.09	11.69 ± 6.46	0.871
	superior	CF SD	3.61 ± 1.91	3.47 ± 2.47	3.26 ± 2.29	0.797
		Δ Imp	12.06 ± 6.50	10.67 ± 8.27	12.08 ± 7.82	0.463
RIPV	anterior	CF SD	3.86 ± 2.61	5.21 ± 2.32	6.07 ± 2.15	0.220
		Δ Imp	11.42 ± 6.04	13.13 ± 4.85	9.10 ± 4.29	0.519
	posterior	CF SD	3.92 ± 2.23	3.33 ± 1.21	2.92 ± 2.28	0.644
		Δ Imp	11.30 ± 7.35	11.01 ± 9.27	9.56 ± 9.05	0.923
	inferior	CF SD	3.67 ± 2.86	4.95 ± 2.58	4.20 ± 1.14	0.591
		Δ Imp	16.69 ± 6.46	12.53 ± 8.58	11.87 ± 3.44	0.325

LSPV, left superior pulmonary vein; LIPV, left inferior pulmonary vein; RSPV, right superior pulmonary vein; RIPV, right inferior pulmonary vein; CF SD, contact force standard deviation; Δ Imp, impedance drop

### Follow-up

No procedural complications occurred during hospitalization.

We prolonged the follow-up time to at least 12 months. A median follow-up time of 16.62 ± 2.61. 1 patients in fixed-curve group had recorded onset of paroxysmal AF at 9 month. 1 patients in conventional aglis group had recorded onset of atrial flutter (AFL) at 11 month. Both patients received a repeated ablation. AF patient was confirmed reconnection of anterior aspect of LSPV. The AFL patient was confirmed as tricuspid isthmus associated typical flutter.

## Discussion

The main findings of this study were as follows: For an ablation apprentice who performed the very first PVI, (1) procedure time was much longer with fixed-curve sheath; (2) steerable sheaths had better catheter stability, but conventional steerable did not reduce fluoroscopic time and might have higher exposure frequency; (3) visualized steerable sheath would reduce both ablation and fluoroscopic time while enhancing catheter stability; (4) instability of ablation catheter was more likely to occur at anterior aspect of LSPV and posterior aspect of RSPV.

Catheter ablation has become an essential method of treating paroxysmal atrial fibrillation, particularly in patients with symptoms and heart failure. New tools have been developed to make the procedure much easier. Steerable and visualized sheaths have been developed that enables the operator to alter the sheath shape and improve manipulation of the catheter around the LA and reduce X-ray exposure. These sheaths have been studied in several clinical trials. The results seemed to be ordinary. A conventional steerable sheath may not shorten PVI and fluoroscopic time in comparison to a fixed-curved sheath [9]. A visualized steerable sheath will reduce fluoroscopic time and increase catheter stability [7]. However, procedures in those trials were performed by highly experienced operators. This may not reflect how steerable sheaths would impact the very first procedure performed by an apprentice, as any expert of ablation has to start as an apprentice.

A success isolation of pulmonary veins is key to a success ablation of PAF. This requires sufficient radio frequency energy delivery at each and every spot with a stable ablation catheter [11]. Achieving stable catheter manipulation requires a perfect understanding of 3D projection of the navigation system and a good cooperation of the sheath and the catheter. For the very first procedure experience, the 3D sense of operator could be distorted by the catheter movement with respiration, even if he had practiced with an ablation simulator. With a fixed-curve sheath and an uni-directional ablation catheter, it could be difficult to reach every position especially anterior aspect of LSPV and posterior aspect of RSPV. Because these positions required the catheter to roll over and bend upward. This research showed that using fixed-curve sheaths resulted in larger CF SD, which reflected catheter instability at anterior aspect of LSPV and posterior aspect of RSPV in particular, and longer procedure time, which implied much difficulty in manipulation. Steerable sheaths dramatically improved maneuverability of the catheter due to its bi-directional curve. Even a fresh new operator was easily attain the superior part of LA with a dorsal curvature [9]. The stability of catheter was also enhanced by a stiffer body of steerable sheaths. At the anterior aspect of LSPV catheter was often unstable due to unintentional catheter slippage on the ridge of the LA appendage, so even with steerable sheaths it would still be a challenge for a novice to maintain sheath stability in the first ablation experience. In our study, CF SD from conventional and visualized steerable sheath group was smaller than the fixed-curve group, but CF SD at anterior aspect of LSPV was still larger than other parts of LA.

A main purpose of introducing steerable sheath which is visible in the CARTO 3 system is to further reduce radiation exposure. For an experienced operator, there is virtually no need of fluoroscope after the sheath and catheter enter LA [12]. Some even take of the heavy lead protective suits for comfort. By contrast, an apprentice may not be able to do so. The reason an apprentice need exposure is to clarify the position of the sheath and the curvature of ablation catheter. Although the conventional steerable sheath is more easily manipulated, it inevitably added two more parameters to the scene, the angle and direction of curvature of the sheath. This could increase the exposure time and frequency of an apprentice. As illustrated in this study, conventional sheaths did not reduce fluoroscopic time and could even increase exposure frequency. With visualized steerable sheaths, it is distinct to perceive real time position, curvature and direction of both sheath and catheter in the X-ray free 3D navigation system, so even in the first ablation of an apprentice, fluoroscopic time and frequency could decrease significantly.

Acute, 3-, and 6-month single procedure success were similar in all three groups. Although 1 patient in the fixed-curve group had AF recurrence, we cannot conclude that steerable sheaths increase success rate because of small sample size. As was reported in previous study, steerable sheath did not result in improved success, but it was much easier to get started for inexperienced operators. However, the cost efficacy should also be considered in daily clinical practice, as both conventional and visualized steerable sheath will increase the cost of each procedure by 10% in our country, but with similar outcome.

## Study limitation

This is an observational study conducted in a single center with a relatively small sized patient and apprentice group. The operators were apprentices and unfamiliar with either sheaths, but the sheath was only one of the factors, as catheter stability and fluoroscopy were also affected by site of transseptal puncture, the anatomy LA, pulmonary veins, and appendage. Because this study focused on the catheter stability and fluoroscopic time during the PVI, it was inadequate to verify the efficacy and safety when using the visualized sheath in this small-sized group. A further prospective large-scale multicenter study is needed to confirm the influence of the use of different sheaths.

## Data Availability

The datasets used and/or analyzed during the current study are available from the corresponding author on reasonable request.
